# Plasma long non-coding RNA BACE1 as a novel biomarker for diagnosis of Alzheimer disease

**DOI:** 10.1186/s12883-017-1008-x

**Published:** 2018-01-09

**Authors:** Liang Feng, Yu-Ting Liao, Jin-Cai He, Cheng-Long Xie, Si-Yan Chen, Hui-Hui Fan, Zhi-Peng Su, Zhen Wang

**Affiliations:** 1 Department of Neurology, The First Affiliated Hospital of Wenzhou Medical University, Wenzhou, 325000 China; 2 Institute of public health management of Wenzhou Medical University, Wenzhou, Wenzhou, 325000 China; 3 Department of Neurosurgery, The First Affiliated Hospital of Wenzhou Medical University, Wenzhou, 325000 China

**Keywords:** Long non-coding RNA, Biomarker, Diagnosis, Alzheimer disease

## Abstract

**Backgrounds:**

Long non-coding RNA (LncRNA) have been reported to be involved in the pathogenesis of neurodegenerative diseases, but whether it can serve as a biomarker for Alzheimer disease (AD) is not yet known.

**Methods:**

The present study selected four specific LncRNA (17A, 51A, BACE1 and BC200) as possible AD biomarker. RT-qPCR was performed to validate the LncRNA. Receiver operating characteristic curve (ROC) and area under the ROC curve (AUC) were applied to study the potential of LncRNA as a biomarker in a population of 88 AD patients and 72 control individuals.

**Results:**

We found that the plasma LncRNA BACE1 level of AD patients was significantly higher than that of healthy controls (*p* = 0.006). Plasma level of LncRNA 17A, 51A and BC200 did not show a significant difference between two groups (*p* = 0.098, *p* = 0.204 and *p* = 0.232, respectively). ROC curve analysis showed that LncRNA BACE1 was the best candidate of these LncRNA (95% CI: 0.553–0.781, *p* = 0.003). In addition, no correlation was found for expression of these LncRNA in both control and AD groups with age or MMSE scale (*p* > 0.05).

**Conclusions:**

Our present study compared the plasma level of four LncRNA between AD and non-AD patients, and found that the level of the BACE1 is increased in the plasma of AD patients and have a high specificity (88%) for AD, indicating BACE1 may be a potential candidate biomarker to predict AD.

**Electronic supplementary material:**

The online version of this article (10.1186/s12883-017-1008-x) contains supplementary material, which is available to authorized users.

## Background

Alzheimer disease (AD), the most predominant type of dementia (50–75%), is a common, progressive and devastating neurodegenerative disease [1]. In 2015, approximate 44 million people worldwide are calculated to have AD or a related dementia disease and the prevalence of AD is expected to triple by 2050 [2]. The disease is clinically chiefly characterized by a profound dysfunction of cognition and progressive deterioration of memory, resulting in loss of autonomy function and ultimately needing full-time medical care [3]. However, until now, no preventive or curative treatment exists for AD, laying an enormous burden on public health and society.

In terms of the mechanism of AD, genetic factors account for most of the variation in the risk of AD, especially in familial AD. Knowledge on genetic variants contributing to amyloid-β (Aβ) processing has evolved enormously throughout the recent years [4]. It started from the discovery of various mutations in Amyloid precursor protein (APP), PSEN 1, PSEN 2 or APOE, which were considered as a cause of autosomal dominant AD and risk factors for both early-onset and late-onset AD patients [5]. More recently, using genome-wide association analyses, about twenty-one additional genetic risk loci for the genetically complex form of AD were detected [6]. Shifting research toward genetic molecular profiling using whole-exome sequencing and transcriptome profiling approaches have led to considerable progress in providing important instructions for complex diseases such as AD [7].

To date, long non-coding RNAs (LncRNA), a novel class of RNAs without encoding-protein capacity have been gained comprehensive attention for their wide range of biological regulatory and modificatory functions [8]. Recently, by the means of genome-wide analyses, plenty of LncRNA have been demonstrated to be involved in the pathogenesis of central nervous disorders and estabilished in different species [9]. Moreover, increasing evidence has suggested that LncRNA play pivotal roles in controlling gene expression and other cellular metabolism processes during developmental and differentiation processes [10]. LncRNA can regulate gene expression at the levels of epigenetic control, transcription, translation and RNA processing and so on [11]. Several recent studies have identified some LncRNA associated with AD, both in human patients and mouse models [12].

What is more, several recent studies have shown that some LncRNA are involved in the occurrence and development of AD [13]. Usually, they located either up or downstream of the enzymes that mediate important pathophysiological processes, such as β-site APP cleaving enzyme-1 (BACE1), and 17A et al. were markedly altered in AD [14, 15]. Among them, BACE1 is essential for the production of the toxic Aβ and the APP processing, which has a major role in AD. Therefore, BACE1 may be a potential biomarker and treatment targets for AD [16, 17]. One previous paper showed that 51A was a fresh LncRNA that maps in an antisense configuration to intron 1 of the neuronal sortilin-related receptor gene (SORL1) gene, which had long been hypothesized to be involved in AD pathogenesis [18]. Notably, 51A is considered to be overexpressed both in vitro models and in the AD brain [19]. Massone et al. reported that 17A would impair GABA signaling, enhance Aβ secretion, and increase the Aβ-42/Aβ-40 ratio [15]. Moreover, 17A is upregulated in AD subjects compared with control group, indicating that it could directly or indirectly take part in the mechanism of AD [20]. Brain cytoplasmic 200 RNA (BC200) is a translational adjustor that targets eukaryotic initiation factor 4A, thereby making for the maintenance of long-term synaptic plasticity [21]. Based on the previous paper, BC200 RNA is upregulated in the AD brain and at least one study reported a downregulation of it [22]. This conflict between multi-studies may be due to the discrepancy in brain regions and varying disease severity, but aberrant BC200 expression in AD is a possibility [13]. Together, these findings provided evidence and support for the potential roles of LncRNA in AD development and progression, and the expression level of LncRNA might serve as biomarkers. Furthermore, LncRNA can be stable level in the plasma and could therefore serve as biomarkers for some diseases. In the present study, we selected several LncRNA that may play important roles in the development of AD, and validated potential AD biomarkers in a moderate-sized cohort to investigated whether LncRNA expression is associated with clinical features and outcomes.

## Methods

### Study subjects

This study was a single-center clinical trial at the First Affiliated Hospital of Wenzhou Medical University. Briefly, the study recruited 160 patients admitted to the Department of Neurology from February 2015 to May 2016, due to clinically diagnosed or suspected AD. The controls were volunteers in the Wenzhou and were never diagnosed before with a central nervous system disorder, including dementia and memory dysfunction, and upon post-mortem histopathological examination did not meet criteria for an AD diagnosis. All the AD patients demonstrated obvious cerebral atrophy by cranial Magnetic Resonance Imaging (MRI) scan and no cerebrovascular lesions. In this study, no cerebrospinal Fluid (CSF) samples were collected to help diagnosis the AD. Meanwhile, disease stage was rated by the Mini-Mental State Examination (MMSE) score, which is a brief cognitive test used widely in clinical practice [23]. The present study was approved by the Ethics Committee of the First Affiliated Hospital of Wenzhou Medical University. In addition, written informed consent was obtained from all patients or their families/relatives in accordance with the Declaration of Helsinki. We obtained the following two groups of samples that were used for this study: AD patients (*n* = 88) and control subjects (*n* = 72) from the Wenzhou Medical Research Institute. In addition, verification of the clinical diagnosis using neuropathology evaluations was finished and reported for all samples.

### Preparation of blood samples

Whole blood was collected from AD subjects and control participants in ethylenediaminetetraacetic acid-coated (EDTA) tubes as an anticoagulant and centrifuged at 1500 g for 15 min (4 °C). Plasma was then transferred to sterile polypropylene tubes on ice and centrifuged again at 3200 g for 15 min (4 °C) to remove platelets. Platelet-free plasma samples were then aliquoted into 1 ml per tube, flash frozen and stored at −80 °C until further analyzed [24].

### RNA isolation and quantitative RT-PCR (Q-PCR)

Total ribonucleic acid (RNA), including LncRNA, was isolated from plasma with the Trizol reagent (Invitrogen, USA). cDNA was generated from total RNA samples using High-Capacity cDNA Reverse Transcription Kits (Takara, Japan). Namely, RNA was reverse transcribed to cDNA in the presence of 50 units of MultiScribe Reverse Transcriptase in a total volume of 20 uL with the following conditions: 37 min at 15 °C, and 5 s at 85 °C. Q-PCR was performed using the ABI 7500 Real-Time PCR System (Life Technologies) according to the supplier’s instructions. As a negative control, template RNA was replaced with PCR-grade water. Calculations of cycle threshold (CT) and difference were analyzed with ABI 7500 Real-Time PCR System.

The primer sequences used in this study were as follows:

- 5-GCAAACGAAGGTTGGTGGTG-3 (forward) and 5-CCCAGCAGTAACCCCCTACT-3 (reverse) for BACE1;
- 5-CTGGGCAATATAGCGAGAC-3 (forward) and 5-TGCTTTGAGGGAAGTTACG-3 (reverse) for BC200.
- 5-CCACCCTGCAACTGACACAT-3 (forward) and 5-GCAAAGGTGCTAATCTTGACTCTTG
-3 (reverse) for 17A;
- 5-TGGGAGAGTCAGCATCTTGAAG-3 (forward) and 5-ACCCTCTCAGTCGTAGAACTTC-3 (reverse) for 51A.

### Statistical analysis

Data are presented as mean ± SD. Differences in LncRNA concentrations between control and AD participants were compared using Student’s t-test or Welch’s t-test for equal or unequal variances; the Mann-Whitney U test was used when the distribution was skewed. Receiver operating characteristic (ROC) and the area under the curve (AUC) was used to test for sensitivity and specificity of each LncRNA. Spearman correlation analyses were used to determine the correlation between LncRNA level and age or MMSE score. Statistical analyses and graph production were performed utilizing SPSS version 13 and Prism version 6.0. *P*-value < 0.05 was considered statistically significant.

## Results

### Clinical basic data

Eighty-eight AD patients and seventy-two control subjects were recruited in this study. The mean age was 67.7 ± 10.0 years, and 35.2% (31/88) were females in AD group, which was much older than that of the control group (51.2 ± 13.0 years, 40.2% females). We found that educational background and MMSE (mini-mental state examination) score in AD patients were significantly lower than that of healthy controls (*p* < 0.05). Moreover, no obvious differences were found in any other variables between two group (*p* > 0.05). The demographic and clinical data of AD Patients and control subjects were summarized in Table 1.

**Table 1:** Clinical characteristics of AD patients and control subjects

	AD patients (%)	Control subjects (%)	*P* value
Total number of subjects	88	72	
Males	57 (64.7)	43 (59.7)	
Females	31 (35.3)	29 (40.3)	> 0.05
Age (years)	67.7 ± 10.0	51.2 ± 13.0	> 0.05
MMSE	13.8 ± 6.9	28.4 ± 1.9	< 0.05
Education background			
Illiteracy	36 (40.9)	7 (9.7)	
literacy	52 (59.1)	69 (90.3)	< 0.05

*MMSE* Mini-Mental State Examination

### Plasma LncRNA in AD patients and control subjects

**Fig. 1:**
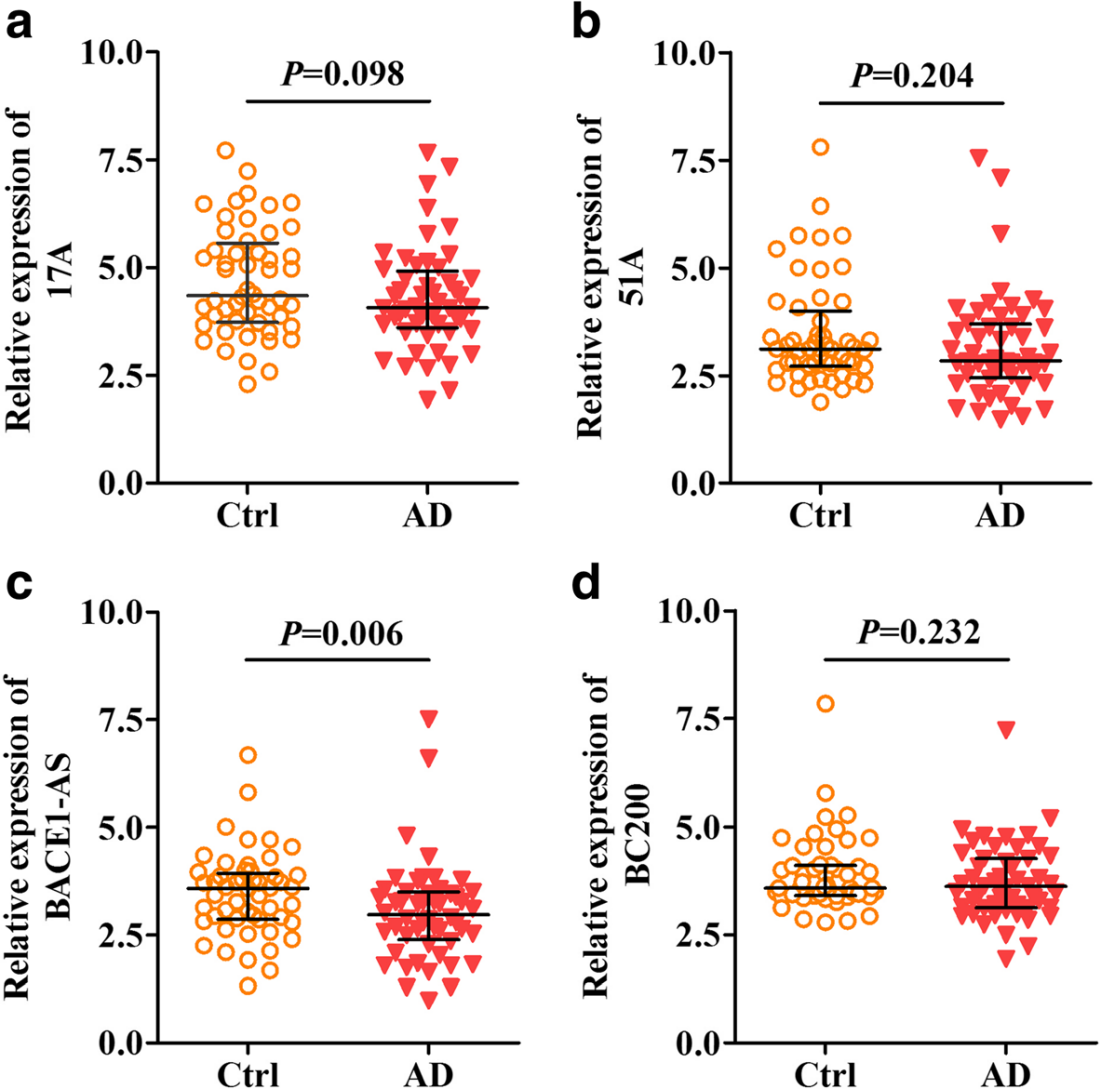
Levels of 17A (**a**), 51A (**b**), BACE1 (**c**) and BC200 (**d**) in plasma of Alzheimer disease (AD) patients and controls. Expression of lncRNAs was expressed relative to their respective level of cel-miR-39. The bar represents median with interquartile range

**Fig. 2:**
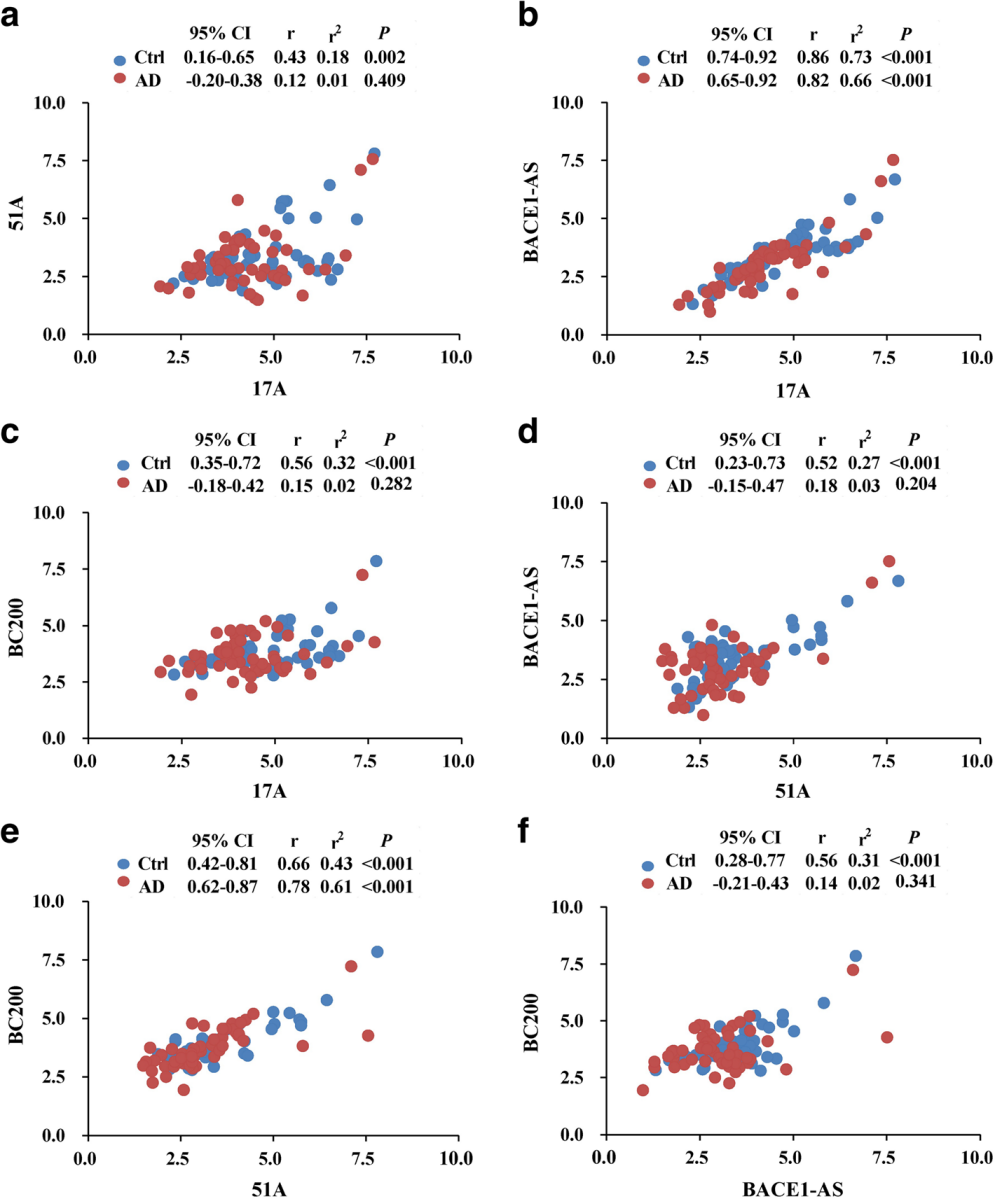
Correlation analyses between 17A, 51A, BACE1 and BC200 in plasma of Alzheimer disease (AD) patients and control (Ctrl) groups. Spearman’s rank correlation coefficient (r) and (r2) along with 95% confidence intervals (CI) and *P* values are listed above each chart. 17A vs 51A (**a**); 17A vs BACE1 (**b**); 17A vs BC200 (**c**); 51A vs BACE1 (**d**); 51A vs BC200 (**e**); and BACE1 vs BC200 (**f**)

To further validate independently the expression of LncRNA 17A, 51A, BACE1 and BC200 in AD plasma, these LncRNA were quantified in plasma samples obtained from a set of 88 AD patients and 72 control individuals. As was shown in Fig. 1, the plasma LncRNA BACE1 level of AD patient was significantly higher than that of healthy controls subjects (*p* = 0.006, Fig. 1). Plasma LncRNA 17A, 51A and BC200 showed no significant difference between two groups (*p* = 0.098, *p* = 0.204, *p* = 0.232, respectively, Fig. 1). Moreover, we further investigated the correlation between LncRNA 17A, 51A, BACE1 and BC200 in plasma of AD patients and control groups, indicating there were mild-moderate positive correlations between LncRNA 17A with LncRNA BACE1 (*r* = 0.82, *p* < 0.001, Fig. 2) and LncRNA 51A with LncRNA BC200 in AD patients (*r* = 0.78, *p* < 0.001, Fig. 2).

### Independent validation of LncRNA expression in AD cohort

**Fig. 3:**
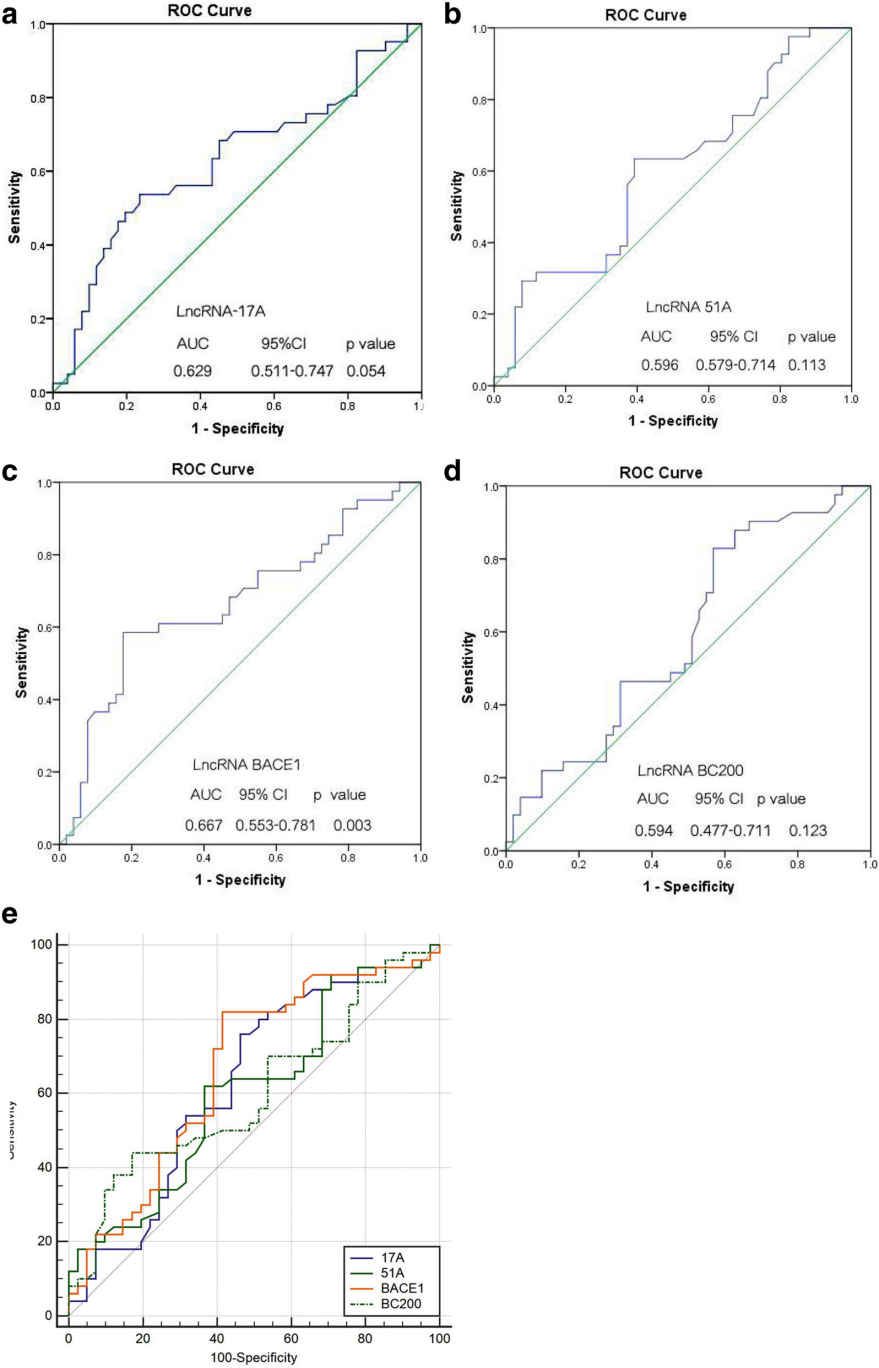
ROC curve analyses of LncRNA 17A (**a**), 51A (**b**), BACE1 (**c**) and BC200 (**d**) for diagnosis of AD in pilot samples. Statistical differences between different ROC curves (**e**). Original set included 88 AD patients and 72 control subjects. AUC = area under the ROC curve, ROC = receiver operating characteristic

To determine the relationship between these LncRNA levels and AD patients, receiver operating characteristic (ROC) analysis was performed in a large group of patients (*n* = 160). It showed an area under the ROC curve (AUC) was 0.629 for 17A (95% CI: 0.511–0.747, *p* = 0.054, Fig. 3a), 0.596 for 51A (95% CI: 0.579–0.714, *p* = 0.113, Fig. 3b), 0.667 for BACE1 (95% CI: 0.553–0.781, *p* = 0.003, Fig. 3c) and 0.594 for BC200 (95% CI: 0.477–0.711, *p* = 0.123, Fig. 3d). These results indicated that BACE1 may be a potential candidate biomarker to predict AD. Meanwhile, comparison of different ROC curves showed no statistical difference (*p* > 0.05, Fig. 3e).

### Relationship between LncRNA and other parameters

To examine whether or not the diagnostic value of LncRNA signature was independent of other parameters for AD patients, including age or MMSE scale, we analyzed the relationship between the LncRNA 17A, 51A, BACE1 and BC200 expression and these parameters by Spearman correlation coefficient. No correlation was found for expression of these LncRNA in both control and CAD group patients with age or MMSE scale (*p* > 0.05), as shown in Tables 2 and 3.

**Table 2:** Correlation analyses between age and expression levels of 17A, 51A, BACE1 and BC200 in plasma of Alzheimer disease (AD) patients and control (Ctrl) groups

LEL	Age (Ctrl)			Age (AD)			Age (AD + Control)		
	r2	CI (95%)	*P* value	r^2^	CI (95%)	*P* value	r^2^	CI (95%)	*P* value
17A	0.03	−0.45~0.01	0.19	0.001	−0.35~0.37	0.83	0.179	−0.356~0.036	0.088
51A	0.012	−0.59~ − 0.10	0.01	0.02	−0.18~0.44	0.31	0.175	−0.360~0.017	0.095
BACE1	0.02	−0.35~0.32	0.30	0.005	−0.24~0.44	0.65	0.217	−0.394~ − 0.004	0.038
BC200	0.08	−0.55~0.02	0.04	0.04	−0.07~0.48	0.11	0.116	−0.313~0.085	0.271

*LEL* LncRNA expression level, *AD* Alzheimer disease, *r* spearman correlation coefficient, *r2* coefficient of determination

**Table 3:** Correlation analyses between MMSE and expression levels of 17A, 51A, BACE1 and BC200 in plasma of Alzheimer disease (AD) patients and control (Ctrl) groups

LEL	MMSE (Ctrl)			MMSE (AD)			MMSE (AD + Control)		
	r2	CI (95%)	*P* value	r^2^	CI (95%)	*P* value	r^2^	CI (95%)	*P* value
17A	0.06	−0.06~0.53	0.12	0.01	−0.21~0.37	0.48	0.262	0.032~0.443	0.012
51A	0.07	−0.10~ − 0.54	0.10	0.02	−0.41~0.12	0.31	0.168	−0.049~0.368	0.105
BACE1	0.05	−0.10~0.51	0.16	0.002	−0.22~0.30	0.79	0.303	0.071~0.480	0.003
BC200	0.02	−0.03~0.50	0.09	0.0001	−0.30~0.30	0.95	0.184	−0.036~0.403	0.079

*LEL* LncRNA expression level, *AD* Alzheimer disease, *r* spearman correlation coefficient, *r2* coefficient of determination

## Discussion

The present study showed that the plasma LncRNA BACE1 level of AD patient was significantly higher than that of healthy controls. Moreover, we found that AUC was 0.667 for BACE1, indicating LncRNA BACE1 can be a potential biomarker for diagnosis of AD patients. Besides, no correlation was found for expression of these LncRNA 17A, 51A, BACE1 and BC200 in both control and CAD group patients with age or MMSE scale.

AD is one of the major neurodegenerative disorders affecting human health worldwide [25]. At present, differential diagnosis between AD and other psychiatric disorders, secondary or primary neurodegenerative dementias associated with early disease onset, is of crucial importance in the prospect of disease-modifying therapies that act on the underlying molecular and pathological processes [26]. However, the current situation is serious and full of challenge. Improved neuroimaging skill and diverse molecular markers of AD have aided diagnosis of AD in the very early stages [27]. However, despite the great endeavors in establishing the contribution of markers to AD, atypical clinical features and disease symptoms still makes its diagnosis a challenge for the clinicians [28]. Moreover, familial aggregation is present in about 25% of all AD cases, the majority being sporadic. The dissemination of genetic testing along with biomarker determinations have prompted a wider recognition of AD in experienced clinical settings [29]. Indeed, genetic testing has prompted a wider recognition of AD in future.

Recently, a growing number of LncRNA have been found to be associated with the prognosis of patients with cancer [30], such as breast cancer, hepatocellular carcinoma and colorectal cancer [31]. Meanwhile, the roles of LncRNA in the development of neurodegenerative diseases are increasingly being studied, including AD [32]. Some data has reported that BACE1 is necessary for amyloid plaques formation and maybe an appropriate drug target for AD treatment [33]. The BACE1 gene surpasses 30 kb and contains nine exons, is a candidate gene for the sporadic AD. Although several results indicated that single nucleotide polymorphisms in exon five of the BACE1 gene related to AD development, clear underlying mechanisms remain hard to identify [34]. Undoubtedly, excessive amyloid β-protein (Aβ) deposition occurs in AD patients. Previous results have shown that familial AD caused by the amyloid precursor protein (APP) mutation, which increases APP split by BACE1 gene, indicating that raised BACE1 activity can result in AD [35]. Therefore, understanding the method to control BACE1 biology function and BACE1 expression may clarify the normal character of BACE1, explicit disease-related underlying mechanisms, and proposal approaches to inhibit BACE1 therapeutically [36]. Whether the BACE1 elevation is actively or passively involved in AD progression is an issue of current investigation. A couple of studies have further demonstrated that BACE1 up-regulation correlated with Aβ pathology and seemed to be more than a passive finish goods of central neurodegeneration disease, whereby Aβ42 deposition in AD results in BACE1 augment, which further boosts Aβ42 expression [37, 38]. Moreover, our finding showed that the plasma LncRNA BACE1 level of AD patient was significantly higher than that of healthy controls, which was consistent with previous theories [39]. More profound comprehending of the molecular and biological mechanisms underlying BACE1 up-regulation in AD will promote the progress of novel therapeutic targets for AD remedy and shed light on the genetic etiology of this catastrophic worldwide disease.

This paper has a number of weaknesses. First, the AD patients and control subjects included in this study are Han Chinese from Wenzhou City. Although a medium size cohort of patients was analyzed in this experiment, it is hard to determine whether the conclusion is applicable to other races and patients from other cities. Second, stability is a basic requirement for any biomarker. We did not investigate the stability of BACE1 in plasma under severe conditions, such as exposure to room temperature and freeze-thaw cycles [40]. Finally, this study did not take the genomics research methods to compare the profile of LncRNA between two groups, such as microarray analysis, due to fund limitation.

## Conclusions

The present study compared the four LncRNA between AD and non-AD patients and found that the level of the BACE1 is increased in the plasma of AD patients. Prospective clinical trials should be carried out to determine the usefulness of BACE1 as a stable plasma biomarker for AD patients.

## Additional file

Additional file 1: Primary data of this clinical trial. This file contains detailed the initial data of all the results in this study. (XLSX 80 kb)

## Abbreviations

AD: Alzheimer disease
APOE: Apolipoprotein E
APP: Amyloid precursor protein
AUC: Area under the ROC curve
Aβ: Amyloid-β
BACE: β-site APP cleaving enzyme-1
BC200: Brain cytoplasmic 200
CSF: Cerebrospinal Fluid
CT: Cycle threshold
EDTA: Ethylenediaminetetraacetic acid-coated
LncRNA: Long non-coding RNA
MMSE: Mini-Mental State Examination
MRI: Magnetic Resonance Imaging
PSEN: Presenilin
ROC: Receiver operating characteristic curve
RT-qPCR: Quantitative real-time Polymerase Chain Reaction
SORL: Sortilin-related receptor gene

## References

[1] Ferri CP, Prince M, Brayne C, Brodaty H, Fratiglioni L, Ganguli M, Hall K, Hasegawa K, Hendrie H, Huang Y, Jorm A, Mathers C, Menezes PR, Rimmer E, Scazufca M (2005). Alzheimer's disease international. Global prevalence of dementia: a Delphi consensus study. Lancet.

[2] Prince M, Bryce R, Albanese E, Wimo A, Ribeiro W, Ferri CP (2013). The global prevalence of dementia: a systematic review and metaanalysis. Alzheimers Dement.

[3] Anand R, Gill KD, Mahdi AA (2014). Therapeutics of Alzheimer's disease: past, present and future. Neuropharmacology.

[4] Janssen JC, Beck JA, Campbell TA, Dickinson A, Fox NC, Harvey RJ, Houlden H, Rossor MN, Collinge J (2003). Early onset familial Alzheimer's disease: mutation frequency in 31 families. Neurology.

[5] Gatz M, Reynolds CA, Fratiglioni L, Johansson B, Mortimer JA, Berg S, Fiske A, Pedersen NL (2006). Role of genes and environments for explaining Alzheimer disease. Arch Gen Psychiatry.

[6] Van Cauwenberghe C, Van Broeckhoven C, Sleegers K (2016). The genetic landscape of Alzheimer disease: clinical implications and perspectives. Genet Med.

[7] Wiseman FK, Al-Janabi T, Hardy J, Karmiloff-Smith A, Nizetic D, Tybulewicz VL, Fisher EM, Strydom A (2015). A genetic cause of Alzheimer disease: mechanistic insights from down syndrome. Nat Rev Neurosci.

[8] Ponting CP, Oliver PL, Reik W (2009). Evolution and functions of long noncoding RNAs. Cell.

[9] Briggs JA, Wolvetang EJ, Mattick JS, Rinn JL, Barry G (2015). Mechanisms of long non-coding RNAs in mammalian nervous system development, plasticity, disease, and evolution. Neuron.

[10] Bertone P, Stolc V, Royce TE, Rozowsky JS, Urban AE, Zhu X, Rinn JL, Tongprasit W, Samanta M, Weissman S, Gerstein M, Snyder M (2004). Global identification of human transcribed sequences with genome tiling arrays. Science.

[11] Hung T, Wang Y, Lin MF, Koegel AK, Kotake Y, Grant GD, Horlings HM, Shah N, Umbricht C, Wang P, Wang Y, Kong B, Langerød A, Børresen-Dale AL, Kim SK, van de Vijver M, Sukumar S, Whitfield ML, Kellis M, Xiong Y, Wong DJ, Chang HY (2011). Extensive and coordinated transcription of noncoding RNAs within cell-cycle promoters. Nat Genet.

[12] Lee DY, Moon J, Lee ST, Jung KH, Park DK, Yoo JS, Sunwoo JS, Byun JI, Shin JW, Jeon D, Jung KY, Kim M, Lee SK, Chu K (2015). Distinct expression of long non-coding RNAs in an Alzheimer's disease model. J Alzheimers Dis.

[13] Wu P, Zuo X, Deng H, Liu X, Liu L, Ji A (2013). Roles of long noncoding RNAs in brain development, functional diversification and neurodegenerative diseases. Brain Res Bull.

[14] Faghihi MA, Zhang M, Huang J, Modarresi F, Van der Brug MP, Nalls MA, Cookson MR, St-Laurent G, Wahlestedt C (2010). Evidence for natural antisense transcript-mediated inhibition of microRNA function. Genome Biol.

[15] Massone S, Vassallo I, Fiorino G, Castelnuovo M, Barbieri F, Borghi R, Tabaton M, Robello M, Gatta E, Russo C, Florio T, Dieci G, Cancedda R, Pagano A (2011). 17A, a novel non-coding RNA, regulates GABA B alternative splicing and signaling in response to inflammatory stimuli and in Alzheimer disease. Neurobiol Dis.

[16] Mulder SD, van der Flier WM, Verheijen JH, Mulder C, Scheltens P, Blankenstein MA, Hack CE, Veerhuis R (2010). BACE1 activity in cerebrospinal fluid and its relation to markers of AD pathology. J Alzheimers Dis.

[17] Dash R, Emran TB, Uddin MM, Islam A, Junaid M (2014). Molecular docking of fisetin with AD associated AChE, ABAD and BACE1 proteins. Bioinformation.

[18] Lee JH, Barral S, Reitz C (2008). The neuronal sortilin-related receptor gene SORL1 and late-onset Alzheimer's disease. Curr Neurol Neurosci Rep.

[19] Ciarlo E, Massone S, Penna I, Nizzari M, Gigoni A, Dieci G, Russo C, Florio T, Cancedda R, Pagano A (2013). An intronic ncRNA-dependent regulation of SORL1 expression affecting Aβ formation is upregulated in post-mortem Alzheimer's disease brain samples. Dis Model Mech.

[20] Gavazzo P, Vassalli M, Costa D, Pagano A (2013). Novel ncRNAs transcribed by Pol III and elucidation of their functional relevance by biophysical approaches. Front Cell Neurosci.

[21] Lin D, Pestova TV, Hellen CU, Tiedge H (2008). Translational control by a small RNA: dendritic BC1 RNA targets the eukaryotic initiation factor 4A helicase mechanism. Mol Cell Biol.

[22] Luo Q, Chen Y (2016). Long noncoding RNAs and Alzheimer's disease. Clin Interv Aging.

[23] Patil RB, Ramakrishnan S (2014). Analysis of sub-anatomic diffusion tensor imaging indices in white matter regions of Alzheimer with MMSE score. Comput Methods Prog Biomed.

[24] Bekris LM, Lutz F, Montine TJ, CE Y, Tsuang D, Peskind ER, Leverenz JB (2013). MicroRNA in Alzheimer's disease: an exploratory study in brain, cerebrospinal fluid and plasma. Biomarkers.

[25] Wimo A, Jönsson L, Bond J, Prince M, Winblad B (2013). The worldwide economic impact of dementia 2010. Alzheimers Dement.

[26] Nordberg A (2015). Dementia in 2014: towards early diagnosis in Alzheimer disease. Nat Rev Neurol.

[27] Dubois B, Feldman HH, Jacova C, Hampel H, Molinuevo JL, Blennow K, DeKosky ST, Gauthier S, Selkoe D, Bateman R, Cappa S, Crutch S, Engelborghs S, Frisoni GB, Fox NC, Galasko D, Habert MO, Jicha GA, Nordberg A, Pasquier F, Rabinovici G, Robert P, Rowe C, Salloway S, Sarazin M, Epelbaum S, de Souza LC, Vellas B, Visser PJ, Schneider L, Stern Y, Scheltens P, Cummings JL (2014). Advancing research diagnostic criteria for Alzheimer's disease: the IWG-2 criteria. Lancet Neurol.

[28] Woolley JD, Khan BK, Murthy NK, Miller BL, Rankin KP (2011). The diagnostic challenge of psychiatric symptoms in neurodegenerative disease: rates of and risk factors for prior psychiatric diagnosis in patients with early neurodegenerative disease. J Clin Psychiatry.

[29] Alberici A, Benussi A, Premi E, Borroni B, Padovani A (2014). Clinical, genetic, and neuroimaging features of early onset Alzheimer disease: the challenges of diagnosis and treatment. Curr Alzheimer Res.

[30] Xie H, Ma H, Zhou D (2013). Plasma HULC as a promising novel biomarker for the detection of hepatocellular carcinoma. Biomed Res Int.

[31] Kogo R, Shimamura T, Mimori K, Kawahara K, Imoto S, Sudo T, Tanaka F, Shibata K, Suzuki A, Komune S, Miyano S, Mori M (2011). Long noncoding RNA HOTAIR regulates polycomb-dependent chromatin modification and is associated with poor prognosis in colorectal cancers. Cancer Res.

[32] Tan L, JT Y, Hu N, Tan L, Non-coding RNA (2013). In Alzheimer's disease. Mol Neurobiol.

[33] Cole SL, Vassar R (2008). The role of amyloid precursor protein processing by BACE1, the beta-secretase, in Alzheimer disease pathophysiology. J Biol Chem.

[34] Clarimón J, Bertranpetit J, Calafell F, Boada M, Tàrraga L, Comas D (2003). Association study between Alzheimer's disease and genes involved in Abeta biosynthesis, aggregation and degradation: suggestive results with BACE1. J Neurol.

[35] Hsiao K, Chapman P, Nilsen S, Eckman C, Harigaya Y, Younkin S, Yang F, Cole G (1996). Correlative memory deficits, Abeta elevation, and amyloid plaques in transgenic mice. Science.

[36] Jiang Y, Rigoglioso A, Peterhoff CM, Pawlik M, Sato Y, Bleiwas C, Stavrides P, Smiley JF, Ginsberg SD, Mathews PM, Levy E, Nixon RA (2016). Partial BACE1 reduction in a Down syndrome mouse model blocks Alzheimer-related endosomal anomalies and cholinergic neurodegeneration: role of APP-CTF. Neurobiol Aging.

[37] Crunkhorn S (2016). Alzheimer disease: BACE1 inhibitor reduces β-amyloid production in humans. Nat Rev Drug Discov.

[38] Zhao J, Fu Y, Yasvoina M, Shao P, Hitt B, O'Connor T, Logan S, Maus E, Citron M, Berry R, Binder L, Vassar R (2007). Beta-site amyloid precursor protein cleaving enzyme 1 levels become elevated in neurons around amyloid plaques: implications for Alzheimer's disease pathogenesis. J Neurosci.

[39] Malkki H (2017). Alzheimer disease: BACE1 inhibition could block CSF tau increase. Nat Rev Neurol.

[40] Vickers KC, Palmisano BT, Shoucri BM, Shamburek RD, Remaley AT (2011). MicroRNAs are transported in plasma and delivered to recipient cells by high-density lipoproteins. Nat Cell Biol.

